# Beneficial Effects of Exogenous Melatonin on Overcoming Salt Stress in Sugar Beets (*Beta vulgaris* L.)

**DOI:** 10.3390/plants10050886

**Published:** 2021-04-28

**Authors:** Pengfei Zhang, Lei Liu, Xin Wang, Ziyang Wang, He Zhang, Jingting Chen, Xinyu Liu, Yubo Wang, Caifeng Li

**Affiliations:** 1 College of Agriculture, Northeast Agricultural University, Harbin 150030, China; agriculture_zpf@163.com (P.Z.); w17745161846@163.com (X.W.); wzy2021neau@163.com (Z.W.); zhanghe@neau.edu.cn (H.Z.); chenjingting2021@163.com (J.C.); agriculture_lxy@163.com (X.L.); 2 Key Laboratory of Mollisols Agroecology, Northeast Institute of Geography and Agroecology, Chinese Academy of Sciences, Changchun 130102, China; liulei@iga.ac.cn

**Keywords:** melatonin, salt stress, sugar beet, photosynthesis, osmolytes, K^+^/Na^+^ homeostasis, antioxidant defense system

## Abstract

Melatonin has been regarded as a promising substance that enhances the abiotic stress tolerance of plants. However, few studies have devoted attention to the role of melatonin in improving salt tolerance in sugar beets. Here, the effects of different application methods (foliar application (100 μM), root application (100 μM), and combined foliar and root application) of melatonin on the morphological and physiological traits of sugar beets exposed to salt stress were investigated. The results showed that melatonin improved the growth of sugar beet seedlings, root yield and sugar content, synthesis of chlorophyll, photosystem II (PS II) activity, and gas exchange parameters under salt stress conditions. Moreover, melatonin enhanced the capacity of osmotic adjustment by increasing the accumulation of osmolytes (betaine, proline, and soluble sugar). At the same time, melatonin increased the H^+^-pump activities in the roots, thus promoting Na^+^ efflux and K^+^ influx, which maintained K^+^/Na^+^ homeostasis and mitigated Na^+^ toxicity. In addition, melatonin strengthened the antioxidant defense system by enhancing the activities of antioxidant enzymes, modulating the ASA-GSH cycle, and mediating the phenylalanine pathway, which removed superoxide anions (O_2_^•−^) and hydrogen peroxide (H_2_O_2_) and maintained cell membrane integrity. These positive effects were more pronounced when melatonin was applied by combined foliar and root application. To summarize, this study clarifies the potential roles of melatonin in mitigating salt stress in sugar beets by improving photosynthesis, water status, ion homeostasis, and the antioxidant defense system.

## 1. Introduction

More sugar must be produced to meet the demands of the growing population, but in recent years, due to limitations of farmland and deterioration of the ecological environment, sugar production has continued to decline [1]. Statistics from the Food and Agriculture Organization (FAO) show that nearly 21% of farmland is affected by salinization worldwide [2]. In addition to the primary salinization caused by natural processes, anthropogenic actions are accompanied by a rapid increase in secondary salinized farmland [3]. Therefore, it is very important to increase sugar production by promoting the utilization of salinized farmland. The sugar beet, whose salt stress tolerance is better than that of most other crops, is one of the major sugar crops [4]. Approximately 35% of sugar products rely on sugar beet worldwide [5]. Thus, growing sugar beets on salinized farmland may be an effective strategy to enhance the production of sugar and the utilization of salinized farmland. For sugar beets, the seedling stage is most vulnerable to salt stress, and salt tolerance gradually increases during growth and development [6].

Under salt stress, osmotic stress, ion toxicity, and oxidative stress caused by excessive accumulation of Na^+^ induce multiple changes in physiological, biochemical, and molecular characteristics, which inhibit plant growth and development [7,8]. Osmotic stress inhibits the division and expansion of cells and the opening of stomata due to restricted absorption of water and nutrients, which is the initial negative effect of salt stress on plants [9,10]. Furthermore, this inhibition is sustained throughout the period of salt stress. Potassium plays an important role in the growth and development of plants [11]. Under salt stress conditions, excessive accumulation of Na^+^ causes K^+^ deficiency, which induces poor growth and premature senescence of plants [12]. The oxidative stress caused by salt in plants is due to the excessive formation of reactive oxygen species (ROS), such as superoxide radicals (O_2_^•−^) and hydrogen peroxide (H_2_O_2_) [13]. ROS can cleave nucleic acids and destroy protein structures, which disturbs gene expression, protein synthesis, and enzymatic activity [14]. On the other hand, lipid peroxidation caused by ROS reduces the selectivity and fluidity of cell membranes [15]. In addition, ROS can degrade photosynthetic pigments and suppress electron transfer, leading to reduced photosynthesis and respiration in plants [16,17].

To safeguard their survival under salt conditions, plants have evolved a defensive system that consists of osmotic adjustment, Na^+^ exclusion, and enhanced antioxidative capacity [18,19]. Osmotic adjustment is produced by the accumulation of osmolytes, such as betaine, proline, and soluble sugar, which maintain the structure of proteins and stability of the cell membrane, reduce the osmotic potential of cells, and provide energy for plants to resist salt stress [20,21]. In a salt environment, the Na^+^/H^+^ antiporters on the plasma membrane (PM) expel Na^+^ from the cytoplasm [22]. At the same time, the gradient of H^+^ caused by H^+^-ATPases and H^+^-PPases on the vacuolar membrane (VM) drives Na^+^ into the vacuole [23]. Therefore, H^+^-pumps are essential for reducing the Na^+^ concentration in the cytoplasm [12]. The antioxidative capacity of plants depends on antioxidant enzyme activities and the formation of antioxidants [24]. The negative effects caused by ROS are alleviated by antioxidative enzymes [25,26]. Antioxidants, such as ascorbic acid (ASA), glutathione (GSH), and secondary metabolites, can also scavenge ROS in the cell [27,28]. However, for plants, the internal defensive system is insufficient to resist salt conditions [29]. Although breeding salt-tolerant varieties is an effective method to overcome salt environments, salt-tolerant genes may influence multiple phenotypes [30], which hinders the breeding of salt-tolerant varieties. Therefore, the application of exogenous substances is a promising strategy that improves the salt tolerance of plants [31,32].

Melatonin widely participates in nature as a ubiquitous molecule, and its biological activity occurs in unicellular organisms, fungi, animals, and plants [33,34]. Melatonin can enhance the tolerance of plants to abiotic stresses, such as low chilling, drought, herbicides, heavy metals, and salt [35,36,37,38,39,40]. Under salt conditions, exogenous application of melatonin regulates a variety of genes and physiological processes involved in resistance to salt stress in plants [41,42,43,44,45,46]. However, these effects of melatonin vary depending on the method of application and the species of plant.

Although melatonin is known to alleviate salt stress damage by regulating physiological and biochemical processes, little information is available regarding the improvement of sugar beet growth under salt stress by applying exogenous melatonin. Therefore, in the current study, our hypotheses were as follows: (1) different methods of melatonin application could enhance the salt stress tolerance of sugar beet seedlings; (2) among these methods, combined root and foliar applications might be optimal; and (3) melatonin could mitigate the damage from salt stress on sugar beets by improving photosynthesis, water status, ion homeostasis, and the antioxidant system. The results would be beneficial for understanding the underlying mechanisms by which melatonin improves the tolerance of sugar beet to salt stress and may provide a promising strategy for promoting sugar production by utilizing salinized farmland.

## 2. Results

### 2.1. Melatonin Improved the Growth of Sugar Beet Seedlings and the Yield and Sugar Content of Roots under Salt Stress

**Figure 1:**
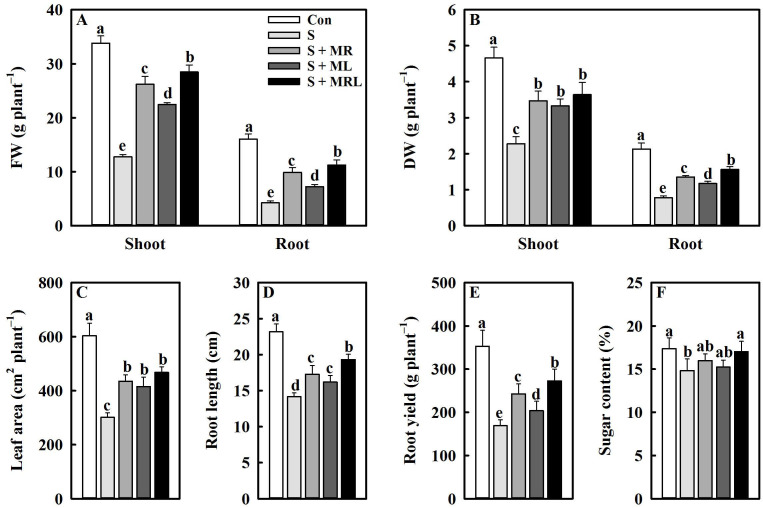
Effect of different methods of melatonin application on growth parameters and root yield and sugar content of sugar beets under salt stress conditions. (**A**) Fresh weight (FW) of shoots and roots, (**B**) dry weight (DW) of shoots and roots, (**C**) leaf area, (**D**) root length, (**E**) root yield, and (**F**) root sugar content. Con, control treatment; S, salt stress treatment; S + MR, salt stress treatment + root application of melatonin; S + ML, salt stress treatment + foliar application of melatonin; S + MRL, salt stress treatment + combined root and foliar applications of melatonin. Data show the means ± standard errors (*n* = 4). Different letters indicate significant differences between treatments according to Duncan’s multiple range test (*p* < 0.05).

Salt stress significantly inhibited the growth of sugar beet seedlings, while the application of melatonin alleviated this inhibition, especially the combined root and foliar applications. Compared with the S (salt stress) treatment, the S + MRL (salt stress treatment + combined root and foliar applications of melatonin) treatment increased the shoot/root FW (fresh weight), shoot/root DW (dry weight), leaf area, and root length of sugar beet seedlings by 15.7/7.0 g·plant^−1^, 1.4/0.8 g·palnt^−1^, 166.5 cm^2^·plant^−1^, and 5.2 cm, respectively (Figure 1A–D).

The yield and sugar content of roots were reduced by salt stress. However, the application of melatonin improved the yield and sugar content of roots under salt stress. Compared with S, S + MR (salt stress treatment + root application of melatonin), S + ML (salt stress treatment + foliar applications of melatonin), and S + MRL increased the root yield by 73.9, 34.8, and 103.8 g plant^−1^, respectively, and the root sugar content by 1.2%, 0.4%, and 2.2%, respectively (Figure 1E,F).

### 2.2. Melatonin Improved the Chlorophyll Content, Gas Exchange Parameters, and Chlorophyll Fluorescence Parameters of Sugar Beet Seedlings under Salt Stress

**Figure 2:**
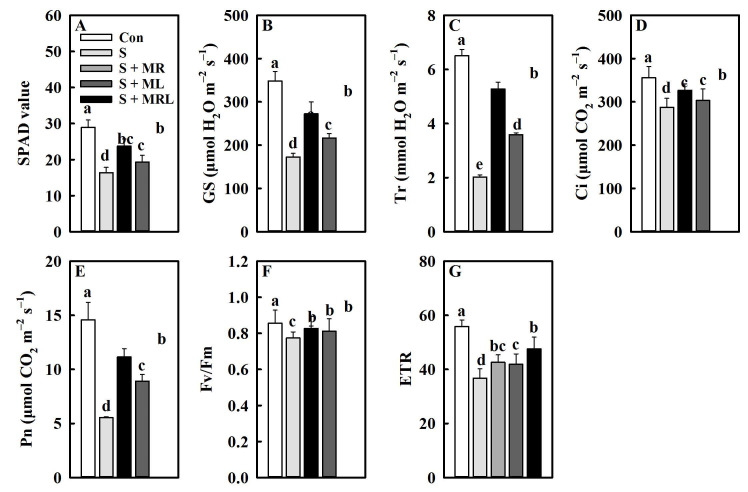
Effect of different methods of melatonin application on chlorophyll content, gas exchange parameters, and chlorophyll fluorescence parameters of sugar beet seedlings under salt stress. (**A**) Chlorophyll content (SPAD value), (**B**) stomatal conductance (GS), (**C**) transpiration rate (Tr), (**D**) intercellular CO_2_ concentration (Ci), (**E**) net photosynthetic rate (Pn), (**F**) maximum quantum yield (Fv/Fm), and (**G**) electron transport rate (ETR) in leaves. Con, control treatment; S, salt stress treatment; S + MR, salt stress treatment + root application of melatonin; S + ML, salt stress treatment + foliar application of melatonin; S + MRL, salt stress treatment + combined root and foliar applications of melatonin. Data show the means ± standard errors (*n* = 4). Different letters in the same column indicate significant differences between treatments according to Duncan’s multiple range test (*p* < 0.05).

Salt stress negatively affects the SPAD value (chlorophyll content) of sugar beet seedlings. However, all methods of melatonin application significantly improved the chlorophyll content under salt stress. Compared with the S treatment, the S + MR, S + ML, and S + MRL treatments increased the chlorophyll content by 3.9, 2.9, and 7.4, respectively (Figure 2A).

Exposure of sugar beet seedlings to salt stress caused significant decreases in all gas exchange parameters. Conversely, the application of melatonin relieved the adverse effects of salt stress on the gas exchange parameters. In particular, the S + MRL treatment significantly increased Gs (stomatal conductance), Tr (transpiration rate), Ci (intercellular CO_2_ concentration), and Pn (net photosynthetic rate) by 99.7 μmol H_2_O m^−2^·s^−1^, 3.3 mmol H_2_O m^−2^·s^−1^, 39.1 μmol CO_2_ m^−2^·s^−1^, and 5.6 μmol CO_2_ m^−2^·s^−1^, respectively, compared with the S treatment. Similar mitigative effects were observed in the S + MR and S + ML treatments (Figure 2B–E).

The Fv/Fm (maximum quantum yield) and ETR (electron transport rate) of sugar beet seedlings were significantly decreased by salt stress, while the application of melatonin increased them. The Fv/Fm and ETR of salt-stressed sugar beet seedlings were increased by 0.05 and 5.9 in the S + MR treatment, by 0.04 and 5.1 in the S + ML treatment, and by 0.06 and 10.9 in the S + MRL treatment, respectively, compared with the non-melatonin treatment (Figure 2F,G).

### 2.3. Melatonin Improved the Accumulation of Osmolytes and the Water Status of Sugar Beet Seedlings under Salt Stress

**Figure 3:**
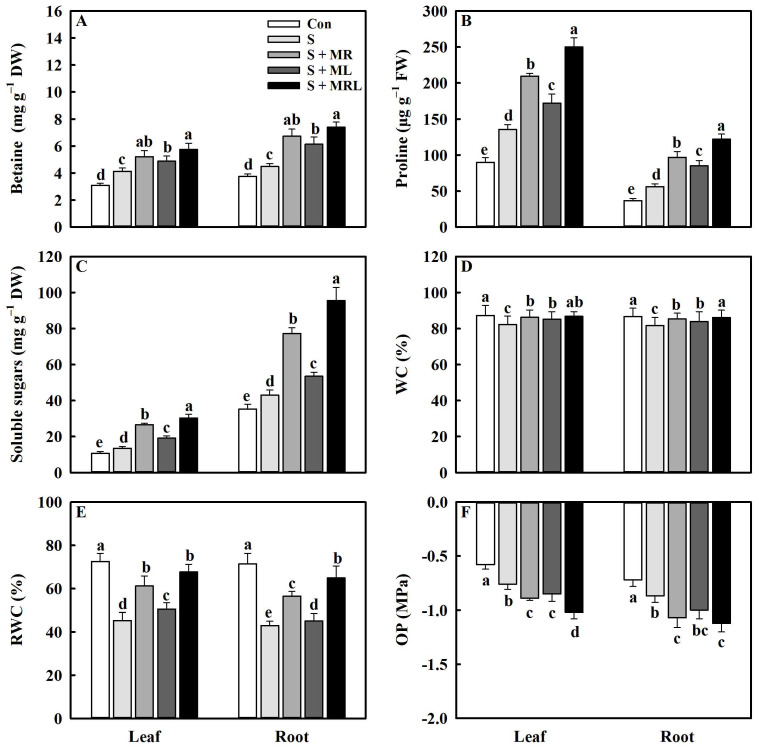
Effect of different methods of melatonin application on the accumulation of osmolytes and water status of sugar beet seedlings under salt stress. (**A**) Betaine content, (**B**) proline content, (**C**) soluble sugar content, (**D**) water content (WC), (**E**) relative water content (RWC), and (**F**) osmotic potential (OP) in leaves and roots. Con, control treatment; S, salt stress treatment; S + MR, salt stress treatment + root application of melatonin; S + ML, salt stress treatment + foliar application of melatonin; S + MRL, salt stress treatment + combined root and foliar applications of melatonin. Data show the means ± standard errors (*n* = 4). Different letters indicate significant differences between treatments according to Duncan’s multiple range test (*p* < 0.05).

Salt stress significantly increased the contents of osmolytes in the leaves and roots of sugar beet seedlings. Nevertheless, the application of melatonin to salt-stressed sugar beet seedlings further increased the contents of osmolytes in leaves and roots, especially the combined root and foliar applications of melatonin. The S + MRL treatment increased the contents of betaine, proline, and soluble sugar by 1.6/2.9 mg g^−1^·DW, 114.4/65.9 mg g^−1^·FW, and 16.7/52.4 mg g^−1^·DW in leaves/roots, respectively, compared to the S treatment (Figure 3A–C).

Under salt stress, the application of melatonin significantly increased WC (water content) and RWC (relative water content) in leaves and roots. Compared with the S treatment, the S + MR, S + ML and S + MRL treatments increased the WC of leaves/roots by 4.0/3.6%, 3.0/2.1%, and 4.6/4.4%, respectively (Figure 3D). Similarly, the RWC of leaves/roots increased by 15.9/13.9%, 5.2/2.5%, and 22.3/21.1%, respectively (Figure 3E). In addition, the application of melatonin to salt-stressed sugar beet seedlings reduced the OP (osmotic potential) in the leaves/roots. The OP of leaves/roots under the S + MR, S + ML, and S + MRL treatments were 17.1/23.0%, 11.8/14.9%, and 34.2/28.7% lower, respectively, than those in the S treatment (Figure 3F).

### 2.4. Melatonin Regulated the Ion Homeostasis of Sugar Beet Seedlings under Salt Stress

**Figure 4:**
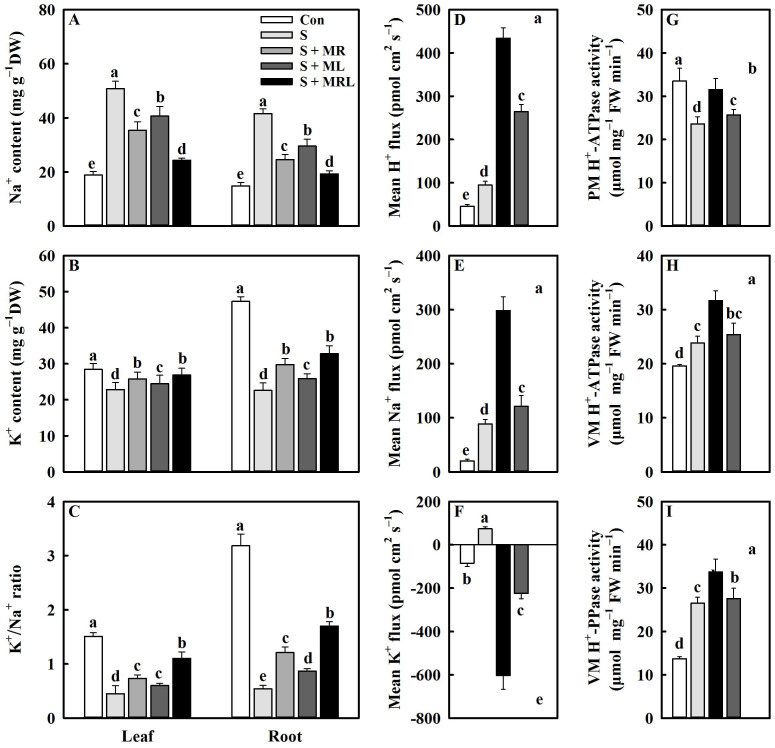
Effect of different methods of melatonin application on ion homeostasis, ion flux, and H^+^-pump activities of sugar beet seedlings under salt stress. (**A**) Na^+^ content, (**B**) K^+^ content, and (**C**) K^+^/Na^+^ ratio in the leaves and roots. The fluxes of (**D**) H^+^, (**E**) Na^+^, and (**F**) K^+^ in the roots; positive values indicate efflux, and negative values indicate influx. The activities of (**G**) PM H^+^-ATPase, (**H**) PM H^+^-ATPase, and (**I**) VM H^+^-PPase in roots. Con, control treatment; S, salt stress treatment; S + MR, salt stress treatment + root application of melatonin; S + ML, salt stress treatment + foliar application of melatonin; S + MRL, salt stress treatment + combined root and foliar applications of melatonin. Data show the means ± standard errors (*n* = 4). Different letters indicate significant differences between treatments according to Duncan’s multiple range test (*p* < 0.05).

Salt stress significantly increases the Na^+^ content in leaves and roots. However, the application of melatonin reduces the Na^+^ content in leaves and roots under salt stress. Relative to the S treatment, the S + MR, S + ML, and S + MRL treatments significantly reduced the Na^+^ content by 15.4/17.1, 10.1/12.0, and 26.4/22.3 mg g^−1^·DW in the leaves/roots, respectively (Figure 4A).

Salt stress treatment caused significant decreases in the K^+^ content and K^+^/Na^+^ ratio in the leaves and roots of the sugar beet seedlings. In contrast, all methods of melatonin application enhanced the K^+^ content and K^+^/Na^+^ ratio in the leaves and roots under salt stress. The maximum increases in K^+^ content and K^+^/Na^+^ ratio in the leaves and roots were observed in the S + MRL treatment (Figure 4B,C).

### 2.5. Melatonin Regulated H^+^, Na^+^, and K^+^ Fluxes and H^+^-Pump Activities in the Roots of Sugar Beet Seedlings under Salt Stress

H^+^ and Na^+^ effluxes in the roots of sugar beet were significantly increased by salt stress. H^+^ and Na^+^ effluxes in the roots of salt-stressed sugar beets were further increased in all melatonin treatments. Relative to the S treatment, the S + MR, S + ML, and S + MRL treatments increased H^+^ effluxes by 276, 169, and 339 pmol cm^2^·s^−1^, respectively, and Na^+^ effluxes by 133, 32, and 209 pmol cm^2^·s^−1^, respectively (Figure 4D,E). In addition, the salt-stressed sugar beets showed slight K^+^ efflux in the roots. All melatonin treatments reversed the K^+^ efflux in the roots under salt stress conditions. Compared with the S + MR and S + ML treatments, the S + MRL treatment was more effective in increasing K^+^ influx (Figure 4F).

Salt stress decreased the PM H^+^-ATPase activity and increased the VM H^+^-ATPase and VM H^+^-PPase activity in the roots. However, the application of melatonin increased the PM H^+^-ATPase activity in the roots under salt stress. Compared with the S treatment, the S + MR, S + ML, and S + MRL treatments significantly increased PM H^+^-ATPase activity by 3.7, 2.1, and 7.9 μmol mg^−1^·FW min^−1^, respectively (Figure 4G). Furthermore, VM H^+^-ATPase and VM H^+^-PPase activity in the roots was further increased under all melatonin treatments, and the most obvious effects were observed in the S + MRL treatment in which VM H^+^-ATPase and VM H^+^-PPase activities in the roots were increased by 7.8 and 7.1 μmol mg^−1^·FW min^−1^ compared to the S treatment, respectively (Figure 4H,I).

### 2.6. Melatonin Inhibited ROS Generation and Promoted Membrane Stability in the Sugar Beets under Salt Stress

**Figure 5:**
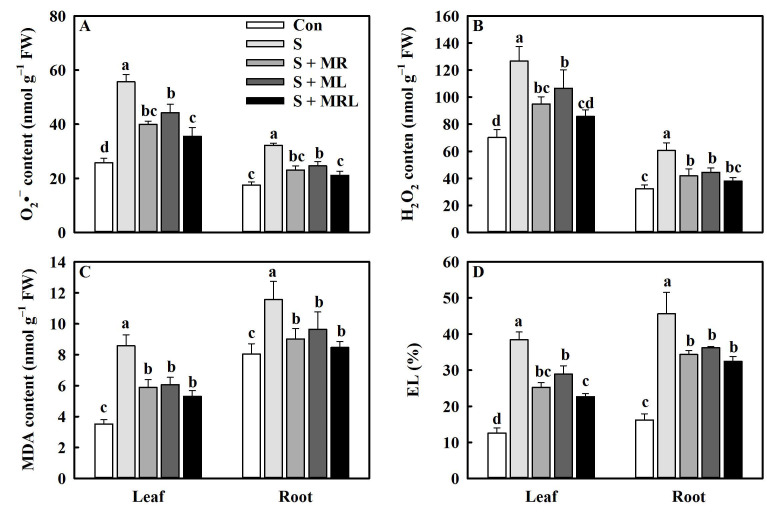
Effect of different methods of melatonin application on ROS generation and membrane stability of sugar beet seedlings under salt stress. (**A**) Superoxide anion (O_2_^•−^) content, (**B**) hydrogen peroxide (H_2_O_2_) content, (**C**) malondialdehyde (MDA) content, and (**D**) electrolyte leakage (EL) in leaves and roots. Con, control treatment; S, salt stress treatment; S + MR, salt stress treatment + root application of melatonin; S + ML, salt stress treatment + foliar application of melatonin; S + MRL, salt stress treatment + combined root and foliar applications of melatonin. Data show the means ± standard errors (*n* = 4). Different letters in the same column indicate significant differences between treatments according to Duncan’s multiple range test (*p* < 0.05).

The application of melatonin to salt-stressed sugar beets reduced the contents of O_2_^−^ (superoxide anion) and H_2_O_2_ (hydrogen peroxide) in the leaves and roots. The S + MR, S + ML, and S + MRL treatments significantly reduced the O_2_^−^ content by 15.7/9.1, 11.4/7.6, and 20.1/11.3 nmol g^−1^·FW, respectively, compared to the S treatment (Figure 5A). Similarly, the H_2_O_2_ content in the leaves/roots was decreased by 31.7/18.6, 19.9/16.1, and 40.9/22.6 nmol g^−1^·FW, respectively (Figure 5B).

Salt stress caused increases in the MDA content and EL in the leaves and roots of sugar beets. However, the malondialdehyde (MDA) content and electrolyte leakage (EL) in the leaves and roots of salt-stressed plants were decreased under all melatonin treatments, especially the S + MRL treatment. The MDA content and EL in the leaves/roots under the S + MRL treatment were 3.3/3.1 nmol g^−1^·FW and 15.8/13.2% lower, respectively, than those under the S treatment (Figure 5C,D).

### 2.7. Melatonin Regulated the Antioxidative System of Sugar Beets under Salt Stress

**Figure 6:**
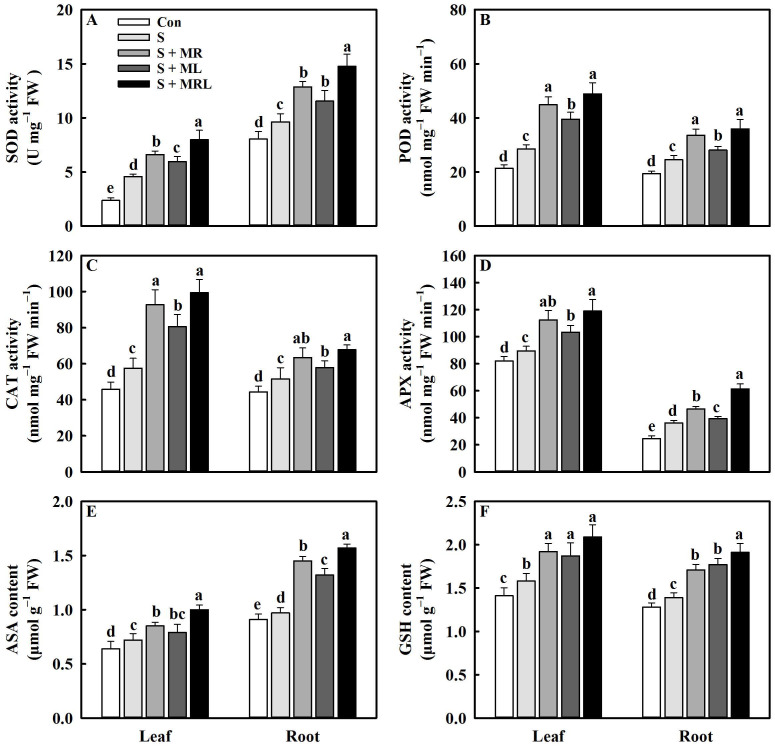
Effect of different methods of melatonin application on antioxidant enzyme activities and antioxidant contents of sugar beet seedlings under salt stress. (**A**) Superoxide dismutase (SOD) activity, (**B**) peroxidase (POD) activity, (**C**) catalase (CAT) activity, (**D**) ascorbate peroxidase (APX) activity, (**E**) ascorbic acid (ASA) content, and (**F**) glutathione (GSH) content in leaves and roots. Con, control treatment; S, salt stress treatment; S + MR, salt stress treatment + root application of melatonin; S + ML, salt stress treatment + foliar application of melatonin; S + MRL, salt stress treatment + combined root and foliar applications of melatonin. Data show the means ± standard errors (*n* = 4). Different letters indicate significant differences between treatments according to Duncan’s multiple range test (*p* < 0.05).

Under salt stress, the activities of SOD (superoxide dismutase), POD (peroxidase), CAT (catalase), and APX (ascorbate peroxidase) in the leaves and roots were increased under all melatonin treatments, and the maximum increases were observed for those under the S + MRL treatments in which the activities of SOD, POD, CAT, and APX in the leaves/roots were increased by 3.4/5.1 U mg^−1^·FW, 20.4/11.4 nmol mg^−1^·FW·min^−1^, 41.2/16.2 nmol mg^−1^·FW·min^−1^, and 29.4/25.2 nmol mg^−1^·FW·min^−1^, respectively, compared with the S treatment (Figure 6A–D). Salt stress also significantly increased the contents of ASA (ascorbic acid, reduced form) and GSH (glutathione, reduced form) in the leaves and roots. Under salt stress, the application of melatonin caused further increases in the contents of ASA and GSH in the leaves and roots. Compared with the S treatment, the S + MR, S + ML, and S + MRL treatments increased the ASA content in leaves/roots by 0.13/0.48, 0.07/0.35, and 0.28/0.6 μmol g^−1^·FW, respectively. Similarly, the GSH content in the leaves/roots increased by 0.34/0.32, 0.29/0.38, and 0.51/0.49 μmol·g^−^^1^·FW, respectively (Figure 6E,F).

### 2.8. Melatonin Increased the Contents of Polyphenolic Compounds in Sugar Beets under Salt Stress

**Figure 7:**
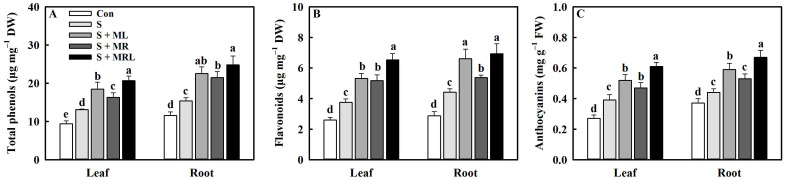
Effect of different methods of melatonin application on the contents of phenolic compounds of sugar beet seedlings under salt stress. (**A**) Total phenol content, (**B**) flavonoid content, and (**C**) anthocyanin content in the leaves and roots. Con, control treatment; S, salt stress treatment; S + MR, salt stress treatment + root application of melatonin; S + ML, salt stress treatment + foliar application of melatonin; S + MRL, salt stress treatment + combined root and foliar applications of melatonin. Data show the means ± standard errors (*n* = 4). Different letters indicate significant differences between treatments according to Duncan’s multiple range test (*p* < 0.05).

Salt stress significantly increased the contents of phenolic compounds in the leaves and roots of sugar beet seedlings. Nevertheless, the application of melatonin to salt-stressed sugar beet seedlings further increased the contents of secondary metabolites in the leaves and roots, especially the combined root and foliar applications of melatonin. The S + MRL treatment increased the contents of total phenols, flavonoids, and anthocyanins by 7.6/9.5 μg·mg^−1^·DW, 2.8/2.5 μg·mg^−1^·DW, and 0.22/0.23 mg·g^−1^·FW in the leaves/roots, respectively, compared to the S treatment (Figure 7A–C).

### 2.9. Melatonin Regulated the Expression Level of Genes in Sugar Beets under Salt Stress

**Figure 8:**
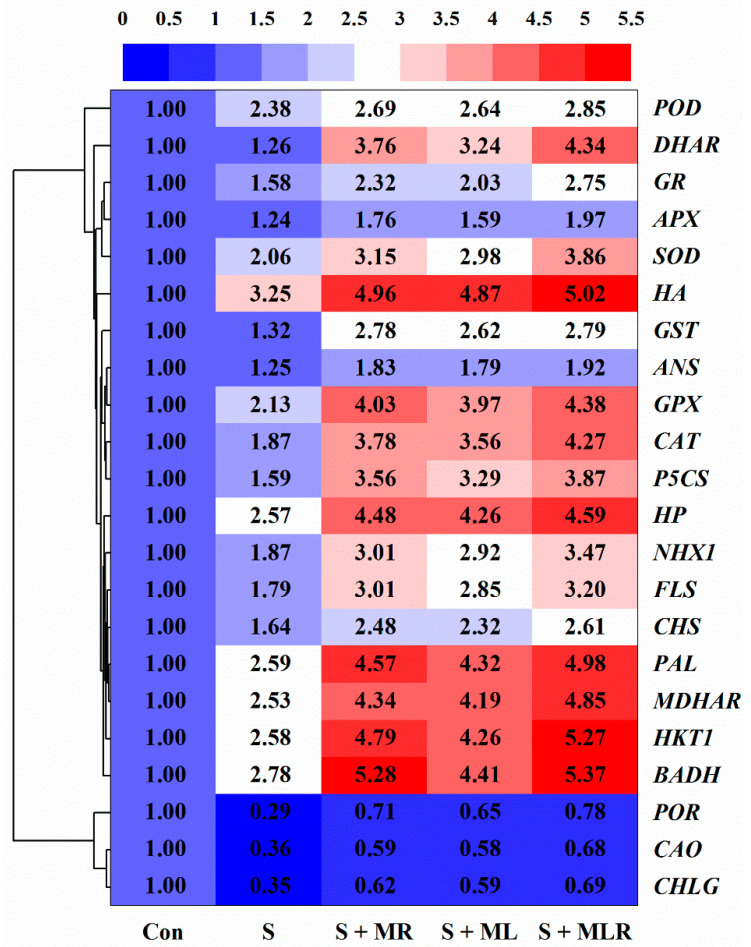
Effect of different methods of melatonin application on the relative expression of genes related to chlorophyll synthesis, osmolyte synthesis, ion transport, H^+^-pumps, antioxidant enzymes, and secondary metabolism synthesis of sugar beet seedlings under salt stress. Con, control treatment; S, salt stress treatment; S + MR, salt stress treatment + root application of melatonin; S + ML, salt stress treatment + foliar application of melatonin; S + MRL, salt stress treatment + combined root and foliar applications of melatonin. The intensity of gene expression extends from blue (low) to red (high).

As shown in Figure 8, salt stress downregulated the expression levels of genes (*CHLG*, *POR*, and *CAO*) associated with chlorophyll synthesis in the sugar beets. Conversely, under salt stress, the expression levels of *CHLG*, *POR*, and *CAO* were upregulated in all melatonin treatments, and the maximum increases were observed in the S + MRL treatment. At the same time, salt stress upregulated the expression levels of betaine biosynthesis-related gene (*BADH*), proline biosynthesis-related gene (*P5CS*), ion transport genes (*HKT1* and *NHX1*), *H^+^-ATPase* (*HA*) gene, *H^+^-PPase* (*HP*) gene, antioxidant enzyme genes (*SOD*, *POD*, *CAT*, *APX*, *MDHAR*, *DHAR*, *GR*, *GST*, and *GPX*), and secondary metabolism-related genes (*PAL*, *CHS*, *FLS*, and *ANS*), while application of melatonin to sugar beets exposed to salt stress further upregulated the expression levels of these genes, especially the combined root and foliar applications of melatonin.

## 3. Discussion

The effects of melatonin application on the morphological, physiological, and biochemical characteristics of sugar beet seedlings under salt stress were illustrated by the results of the current study. To our knowledge, this is the first report showing that different methods of melatonin application improve the tolerance of sugar beet seedlings to salt stress.

In the current study, the biomass and morphology of sugar beets were obviously negatively affected by salt stress. This is because excessive accumulation of Na^+^ in cells caused by salt stress induces osmotic stress, ion toxicity, and oxidative damage, leading to inhibition of cell division and expansion, which causes poor growth of plants [8]. However, all methods of melatonin application increased fresh weight, dry weight, leaf area, root length, and yield under salt stress (Figure 1A–E), suggesting that application of melatonin effectively improves the growth of sugar beets under salt stress. Similar to our results, Arnao and Hernandez-Ruiz [34] reported that melatonin can improve the resistance of various plants to adverse environments. In addition, the application of melatonin increased the root sugar content of sugar beets exposed to salt stress (Figure 1F). A potential reason for this positive effect may be that the application of melatonin is beneficial for the Calvin cycle and the activities of enzymes involved in sucrose synthesis [47], thus increasing the sugar content in the cytoplasm of sugar beets under salt stress conditions.

The photosynthesis of plants can be significantly inhibited by salt stress [16]. The results of the present study support this conclusion, as the SPAD value, gas exchange parameters, and chlorophyll fluorescence parameters of the sugar beets were significantly reduced under salt stress (Figure 2A–G). The negative effects of salt stress on the chlorophyll content of the sugar beets were alleviated by applying melatonin. This result agrees with a study by Kamiab [48], who reported that melatonin protects the ultrastructure of chloroplasts and improves the activity of chlorophyll synthase to promote the formation of chlorophyll in pistachios under salt stress. Furthermore, the application of melatonin to sugar beets exposed to salt stress increased the relative expression of *CHLG*, *POR*, and *CAO* genes (Figure 8), which provides direct support for this conclusion. Photosystem II (PSII), whose activity can be reflected by chlorophyll fluorescence parameters, is an important part of the photosystem [49]. The increase in chlorophyll content promotes the capture and utilization of light energy by photosynthetic organs, which provides a foundation for maintaining PSII and PSI activity [16]. In addition, the application of melatonin may alleviate the damage caused by salt stress in photosynthetic organs, thereby improving electron transfer in PS II [50]. These findings may explain why the Fv/Fm and ERT under melatonin treatment were higher than those under non-melatonin treatment with salt stress (Figure 2F,G).

It has been recognized that the accumulation of osmolytes is an effective strategy for plants to combat osmotic stress caused by salt stress [32]. In our study, the application of melatonin further increased the contents of osmolytes, including betaine, proline, and soluble sugar (Figure 3A–C), and upregulated the expression of genes, including *BADH* and *P5CS* (Figure 8), suggesting that melatonin plays an important role in the resistance of sugar beets to salt stress. Previous studies have demonstrated that the accumulation of these osmolytes induced by melatonin reduces the osmotic potential of cells, which improves osmotic adjustment and thus enhances the water content of plants under adverse environments [48,51]. The results of the current study directly support this conclusion since melatonin-treated sugar beets exhibited increases in WC and RWC and decreases in OP compared with non-treated plants under salt stress (Figure 3D–F). The other reason for the improvement of water status under salt stress may be that melatonin improves root growth and aquaporin activity, which enhances water absorption and transport [52]. In addition, a high level of water content induces the opening of leaf stomata, thus enhancing the exchange of H_2_O and CO_2_ between leaves and the air and ultimately leading to the improvement of photosynthesis in plants [44]. This may explain why the application of melatonin increased Gs, Tr, Ci, and Pn in sugar beets under salt stress (Figure 2B–E).

The depolarization of PM induced by salt stress opens non-selective cation channels and K^+^ outward rectifying channels, leading to excessive Na^+^ influx into the cells and K^+^ efflux from the cells, which causes ion imbalance and Na^+^ toxicity in plants [8,12]. Similar results were obtained in the current study. Salt stress increased the Na^+^ content and decreased the K^+^ content in the sugar beets, leading to a decrease in the K^+^/Na^+^ ratio (Figure 4A–C). However, all methods of melatonin could alleviate these negative effects, indicating that melatonin may regulate ion homeostasis and mitigate ion toxicity in sugar beets under salt stress conditions. Repolarization of PM is essential for maintaining ion homeostasis in plants [22]. Under salt stress, the repolarization of PM relies on the activity of the H^+^-pump that transports H^+^ outside the PM [22,42]. The current study showed that the application of melatonin increased the activity of PM H^+^-ATPase and H^+^ efflux from the roots of sugar beets under salt stress (Figure 4D,G). At the same time, the enhancement of the Na^+^ efflux and the reversion of the K^+^ efflux were observed in the roots under melatonin combined with salt stress treatment (Figure 4E,F). These results imply that melatonin may maintain the ion homeostasis of sugar beets under salt stress by regulating the activity of PM H^+^-ATPase. Previous studies have revealed that high-affinity K^+^ transporters can drive K^+^ into the cytoplasm and Na^+^/H^+^ antiporters can expel Na^+^ from the cytoplasm [12,23]. In the current study, the application of melatonin upregulated the relative expression of the *HKT1* and *NHX1* genes in the roots of sugar beets exposed to salt stress (Figure 8), which also confirms that melatonin plays an important role in maintaining ion homeostasis in plants under salt stress conditions [42]. Another possible reason for this positive effect may be that melatonin regulates polyamine metabolism and thus promotes the production of NO, which mediates the physiological processes related to the regulation of ion homeostasis [39]. In addition, detoxification of cells in plants is closely related to compartmentalization of Na^+^ [15]. VM H^+^-ATPase and VM H^+^-PPase establish the H^+^ gradient of the VM to drive Na^+^ from the cytoplasm into the vacuole, which protects the organelles from salt stress to maintain various physiological processes [12]. In the current study, the application of melatonin further increased the activities of VM H^+^-ATPase and VM H^+^-PPase and upregulated the expression of the genes (*HA* and *HP*) encoding them in roots under salt stress (Figure 4H,I and Figure 8), suggesting that melatonin may promote Na^+^ compartmentalization to alleviate toxicity in sugar beets caused by salt stress.

Under salt stress conditions, overproduction of ROS caused by excessive accumulation of Na^+^ induces lipid peroxidation, thus destroying the stability and integrity of PM, which disrupts physiological and biochemical processes in plants [15]. The degree of lipid peroxidation can be measured by the MDA content and EL [7]. In the current study, salt stress increased the contents of O_2_^−^ and H_2_O_2_, accompanied by increases in MDA content and EL (Figure 5A–D). However, regardless of the method of application, melatonin reduced the above parameters under salt stress, suggesting that melatonin may mitigate the oxidative damage caused by salt stress in sugar beets. As the first line of defense against oxidative stress, antioxidant enzymes can effectively remove ROS [19,25]. The current study showed that melatonin further enhanced the activities of SOD, POD, and CAT and positively regulated the expression of the genes encoding them in sugar beets exposed to salt stress (Figure 6A–C and Figure 8). Similar findings were presented by Bahcesular et al. [31] and Ren et al. [44], who reported that melatonin induces scavenging of ROS in salt-stressed basil and maize by enhancing antioxidant enzyme activity. The ASA-GSH cycle can cooperate with antioxidant enzymes to eliminate ROS in plants [12]. Melatonin increases APX activity by balancing ASA and GSH pools, which contributes to maintaining redox homeostasis and reducing oxidative damage in cells [38]. Consistent with the previous result, the current study also showed that all methods of melatonin application positively regulated APX activity along with the contents of ASA and GSH in sugar beets exposed to salt stress (Figure 6D–F). Under salt stress, upregulation of genes (*APX*, *MDHAR*, *DHAR*, *GR*, *GST*, and *GPX*) involved in the ASA-GSH cycle were observed under melatonin treatment (Figure 8), which further confirms the positive effects of melatonin on the regulation of the ASA-GSH cycle in salt-stressed sugar beets. On the other hand, polyphenolic compounds are secondary metabolites that are directly involved in the scavenging of ROS in various organelles and are regarded as the second line of defense against oxidative stress [38]. Under environmental stresses, when antioxidant enzymes are insufficient to eradicate ROS, the phenylalanine pathway is triggered for the generation of polyphenolic compounds, including flavonoids and anthocyanins [53]. In addition, polyphenolic compounds can chelate Fe^2+^, which interferes with the Fenton reaction and thus inhibits the formation of ROS [54]. Further increases in the contents of total phenols, flavonoids, and anthocyanins were found in all melatonin treatments under salt stress (Figure 7A–C), suggesting that melatonin may improve the antioxidant defense system of sugar beets by modulating the phenylalanine pathway. To confirm this conjecture, we quantified the expression levels of genes (*PAL*, *CHS*, *FLS*, and *ANS*) associated with the phenylalanine pathway. The expression of these genes was also significantly upregulated in salt-stressed sugar beets in all melatonin treatments (Figure 8).

## 4. Materials and Methods

### 4.1. Plant Materials and Growing Conditions

The pot experiment was carried out in a glass greenhouse with a day/night temperature of 25/18 ± 2 °C and relative humidity of 65% to 75%. Pelleted seeds of sugar beet (*Beta vulgaris* L. cv. KWS0143) purchased from Kengfeng Seed Co. Ltd. (Harbin, Heilongjiang, China) were used in this work. Five seeds were sown in each pot (30 cm diameter and 35 cm height) containing 10 kg of soil. The properties of the soil are shown in Table S1. After sowing, each pot was irrigated with 2 L of half-strength Hoagland solution (HHS) every 7 days during the experiment.

### 4.2. Treatments and Experimental Layout

The experiments were carried out in a randomized complete block design with four replicates for each treatment. The treatments were as follows: (i) control treatment (Con), seedlings were irrigated with HHS; (ii) salt stress treatment (S), seedlings were irrigated with HHS containing 600 mM NaCl; (iii) salt stress treatment + root application of melatonin (S + MR), seedlings were irrigated with HHS containing 600 mM NaCl and 100 μM melatonin; (iv) salt stress treatment + foliar application of melatonin (S + ML), seedlings were irrigated with HHS containing 600 mM NaCl and sprayed with 100 μM melatonin solution; and (v) salt stress treatment + combined root and foliar applications of melatonin (S + MRL), seedlings were irrigated with HHS containing 600 mM NaCl and 100 μM melatonin and sprayed with 100 μM melatonin solution.

Salt stress treatment was synchronized with different methods of melatonin application at 21, 28, and 35 days after sowing (DAS). Salt stress treatment or root application of melatonin was performed by adding NaCl or melatonin to the HHS. Foliar application of melatonin was carried out as follows. Before the seedlings were sprayed, the soil surface was covered with a plastic film to prevent the melatonin solution from dropping on the soil, and then, each pot was sprayed with 20 mL of melatonin solution to ensure that each leaf was subjected to melatonin solution. The desired NaCl concentration was based on preliminary experiments, in which sugar beet seedlings were continuously irrigated with HHS containing 200, 400, 600, 800, and 1000 mM NaCl, respectively, every 7 days. After three irrigations, withered seedlings were observed at 800 and 1000 mM NaCl. The melatonin concentration was selected according to a previous study, which reported that 100 μM melatonin effectively enhances abiotic stress tolerance in various plants [55].

The relevant parameters were measured at 40 DAS. At the same time, fresh samples from the topmost fully developed leaves and roots were collected from each treatment and stored at −80 °C until physiological and biochemical assessments were performed. Then, seedlings were thinned to one seedling per pot to determine the yield and sugar contents of the roots at harvest.

### 4.3. Determination of Morphological Parameters and Root Yield and Sugar Content

Ten sugar beet seedlings were randomly selected from each treatment at 40 DAS to determine the growth parameters. The seedlings were washed to clear away the soil particles and wiped with a paper towel, and then, FW was recorded. Leaf area was measured using a leaf area meter (LI-3000C, LI-COR, Lincoln, NE, USA). Root length was determined using a ruler. Then, seedlings were oven-dried at 75 °C to record the dry weight DW.

Five sugar beets were randomly selected from each treatment at 145 DAS. The roots were washed with running water and weighed to measure the yield. Then, the sugar contents of the roots were determined using a refractometer (MPR E-Scn, EMC Inc., Umatilla, FL, USA).

### 4.4. Determination of Chlorophyll Content and Gas Exchange and Chlorophyll Fluorescence Parameters

Four sugar beet seedlings were randomly selected from each treatment to determine chlorophyll content and gas exchange and chlorophyll fluorescence parameters. The chlorophyll contents of the topmost fully expanded leaves were measured by using a chlorophyll meter (SPAD-502, Konica Minolta, Tokyo, Japan).

The Gs, Tr, Ci, and Pn were determined using a portable photosynthetic system (GFS-3000, WALZ, Effeltrich, Germany). The photosynthetically active radiation, relative humidity, temperature, and CO_2_ concentration in the leaf chamber were set to 800 µmol·m^−2^·s^−1^, 75%, 27 °C, and 600 µmol·mol^−1^, respectively. The topmost fully developed leaves were measured after the values of relative humidity and CO_2_ concentration were stabilized.

The chlorophyll fluorescence parameters were determined using a chlorophyll fluorometer (PAM–2500, WALZ, Effeltrich, Germany). After 30 min of dark adaptation, the topmost fully expanded leaves were measured. The F_v_/F_m_ and ERT were automatically obtained by the instrument.

### 4.5. Determination of Osmolyte Contents

The betaine content was determined according to the method described by Chung et al. [56]. Briefly, 2 g of powdered dried samples were mixed with 10 mL of methanol and then heated in boiling water for 1 h. After centrifugation (12,000× *g*, 20 min), 5 mL of supernatant was mixed with 10 mL of 2.5% (*w*/*v*) reinecke salt and 10 mL of 70% (*v*/*v*) acetone. The absorbance was recorded at 525 nm by using a spectrophotometer (UV-2450, Shimadzu Corp., Kyoto, Japan).

The proline content was assessed according to Bates et al. [57]. In short, 0.5 g of fresh samples were homogenized in 5 mL of 3% sulfosalicylic acid and then heated in boiling water for 10 min. After centrifugation (5000× *g*, 15 min), 2 mL of supernatant were mixed with 2 mL of acetic acid and 2 mL of 2.5% (*w*/*v*) ninhydrin. The reaction mixture was heated in boiling water for 1 h, after which the reaction was terminated by adding 4 mL of toluene. The absorbance was determined at 520 nm.

The content of soluble sugar was measured following the procedure described by Spiro [58]. Dry samples (0.1 g) were extracted with 5 mL of distilled water at 100 °C for 10 min and then centrifuged at 10,000× *g* for 5 min. The supernatant was mixed with 0.5 mL of 3% (*w*/*v*) anthrone and 5 mL of sulphuric acid. The mixture was heated in boiling water for 5 min and then cooled. The absorbance was measured at 630 nm.

### 4.6. Determination of Water Status Parameters

The samples (topmost fully developed leaves and root tips) were collected and immediately weighed to FW. Then, the samples were immersed in distilled water for 12 h, and the TW (turgid weight) was measured. After the samples were dried at 80 °C for 24 h, the DW was determined. The WC and RWC were calculated as follows [59]:

WC=(FW − DW)/DW × 100

RWC=(FW − DW)/(TW − DW) × 100

OP was measured according to the method described by Yuan et al. [60]. First, 5 g of fresh samples were frozen in liquid nitrogen for 5 min and then centrifuged at 5000× *g* for 30 min to collect the cell sap. The OP was measured with a dew point microvolt meter (Vapro 5520, Wescor, Logan, UT, USA).

### 4.7. Determination of Na^+^ and K^+^ Contents

The Na^+^ and K^+^ contents were determined following the method of Storey [61]. Dry samples (0.2 g) were digested with a mixture of nitric acid and perchloric acid (3:1, *v*/*v*) at 270 °C until the mixture was clarified. The Na^+^ and K^+^ contents were measured using a flame photometer (BWB-XP, BWB Technologies Ltd., Newbury, Berkshire, UK).

### 4.8. Determination of H^+^, Na^+^, and K^+^ Fluxes and H^+^-Pump Activities of the Roots

H^+^, Na^+^, and K^+^ fluxes were assessed by using an NMT system (NMT-100, Younger, Amherst, MA, USA) according to the method of Kong et al. [62]. Roots were washed with deionized water and then immersed in 10 mL of measuring solution containing 0.1 mM KCl, 0.1 mM MgCl_2_, 0.1 mM CaCl_2_, 0.5 mM NaCl, 0.2 mM Na_2_SO_4_, and 0.3 mM 2-(N-morpholino) ethanesulfonic acid. The H^+^, K^+^, and Na^+^ fluxes at 750 μm from the root apex were measured for a duration of 15 min after the flux rate was stabilized.

Fresh samples (0.1 g) were homogenized in 1 mL of phosphate buffer (pH 7.4) and centrifuged at 5000× *g* for 20 min at 4 °C. Then, microcapsules of PM and VM were obtained according to the method of Yan et al. [39]. The activities of PM H^+^-ATPase, VM H^+^-ATPase, and VM H^+^-PPase were measured using ELISA kits (R&D Systems, Minneapolis, MN, USA).

### 4.9. Determination of Reactive Oxygen Species (ROS) Generation

The O_2_^•−^ level was evaluated using the method described by Zhang et al. [63]. Fresh samples (0.2 g) were homogenized in 1 mL of 0.05 M phosphate buffer (pH 7.8) and centrifuged at 12,000× *g* for 10 min at 4 °C. Then, 0.5 mL of the supernatant was added to 1 mL of 10 mM hydroxylamine hydrochloride and incubated at 25 °C for 30 min. Then, the solution was mixed with 1 mL of 17 mM sulfanilamide and 1 mL of 7 mM naphthylamine. After the second incubation (25 °C, 10 min), the absorbance was measured at 530 nm.

The H_2_O_2_ content was determined following the procedure of Velikova et al. [64]. Fresh samples (0.2 g) were homogenized in 1 mL of 0.1% (*w*/*v*) trichloroacetic acid and centrifuged at 12,000× *g* for 10 min. Then, 0.5 mL of supernatant was mixed with 2 mL of 1 M potassium iodide and 0.5 mL of phosphate buffer. After 30 min of incubation in the dark, the absorbance was recorded at 390 nm.

### 4.10. Determination of MDA and EL

The MDA content was measured according to the thiobarbituric acid (TBA) method [65]. Fresh samples (0.5 g) were homogenized in 5 mL of 5% (*w*/*v*) trichloroacetic acid. The homogenate was centrifuged at 5000× *g* for 10 min. Then, 2 mL of supernatant were mixed with 2 mL of 0.67% (*w*/*v*) TBA and heated at 100 °C for 30 min. Afterwards, the mixture was rapidly cooled to stop the reaction. The absorbance was measured at 452, 532 and 600 nm.

The EL was determined using the method of Dionisio-Sese and Tobita [66]. First, 2 g of fresh samples were cut into 2-mm segments and transferred into a tube containing 20 mL of deionized water. After 24 h of incubation at room temperature, the initial electrical conductivity (EC_1_) was measured using an electrical conductivity meter (A-212, Thermo Fisher Scientific, Chicago, IL, USA). Then, the tube was heated at 100 °C for 20 min and cooled. Afterwards, the second electrical conductivity (EC_2_) was measured. The EL (%) was calculated using the following formula:

$${\mathrm{EL}=\mathrm{EC}}_{1}{/\;\mathrm{EC}}_{2}\;\times \;100$$

### 4.11. Determination of Antioxidant Enzyme Activities

To collect the enzyme extract, 1 g of fresh sample was homogenized in 2 mL of phosphate buffer (pH 7.0) using a pre-cooled mortar and centrifuged at 12,000× *g* for 20 min at 4 °C. The supernatant was used to measure the activities of SOD, POD, CAT, and APX.

SOD activity was calculated according to the method described by Giannopolitis and Ries [67]. First, 50 μL of enzyme extract were mixed with 3 mL of reaction solution containing 50 mM phosphate buffer (pH 7.8), 13 mM methionine, 75 μM nitro-blue tetrazolium, 10 μM ethylenediaminetetraacetic acid disodium salt, and 2 μM riboflavin. After 30 min of illumination at 4000 lx, the absorbance was recorded at 560 nm. One unit (U) of SOD activity represented the amount of enzyme required for 50% inhibition of the photochemical reduction of nitro-blue tetrazolium.

The POD activity was determined following the method of He et al. [68]. The reaction mixture consisted of 2.9 mL of 50 mM phosphate buffer (pH 5.5), 1 mL of 0.6 M hydrogen peroxide, 1 mL of 50 mM guaiacol, and 0.1 mL of enzyme extract. The reaction mixture was incubated at 37 °C for 15 min. Afterwards, the reaction was terminated by adding 2 mL of 20% (*v*/*v*) trichloroacetic acid. The change in absorbance caused by oxidation of guaiacol was measured at 470 nm.

The CAT activity was measured using the method described by Nabih et al. [69]. First, 50 μL of enzyme extract were mixed with 0.5 mL of 0.1 M hydrogen peroxide and 2 mL of phosphate buffer (pH 7.0). The change in absorbance due to the decomposition of hydrogen peroxide was measured at 240 nm.

The APX activity was evaluated following the method of Fielding and Hall [70]. First, 100 μL of enzyme extract were mixed with 3 mL of reaction solution containing 50 mM phosphate buffer (pH 7.0), 0.1 mM ethylenediaminetetraacetic acid disodium salt, 0.3 mM ascorbic acid, and 60 μM hydrogen peroxide. The decrease in absorbance caused by the oxidation of ascorbic acid was measured at 290 nm.

### 4.12. Determination of Antioxidant Contents

The reduced ASA content was determined using the method of Logan et al. [71]. First, 1 g of fresh sample was homogenized in 5 mL of 5% (*v*/*v*) perchloric acid, followed by centrifugation at 20,000× *g* for 10 min. Then, 1 mL of supernatant was mixed with 5 mL of reaction solution, which consisted of 3 mL of ethanol, 0.5 mL of 0.4% (*v*/*v*) phosphoric acid, 1 mL of 15 mM bathophenanthroline, and 0.5 mL of 2 mM ferric chloride. After 90 min of incubation at 30 °C, the absorbance was measured at 534 nm.

The reduced GSH content was measured following the method described by Griffith [72]. Fresh samples (0.2 g) were homogenized in 2 mL of 5% (*w*/*v*) metaphosphoric acid using a pre-cooled mortar. The homogenate was centrifuged at 12,000× *g* for 10 min. Then, 2 mL of the supernatant were mixed with 4 mL of 0.2 M phosphate buffer (pH 7.0) and 0.4 mL of 5,5′-dithiobis-(2-nitrobenzoic acid) and incubated in a shaker at 30 °C for 5 min. The glutathione content was measured at 412 nm.

### 4.13. Determination of Contents of Phenolic Compounds

The total phenol content was determined according to the method of Singleton and Rossi [73]. In short, 2 g of fresh samples were immersed in 20 mL of distilled water and heated in boiling water for 15 min. After centrifugation (5000× *g*, 20 min), 1 mL of supernatant was mixed with 3 mL of 0.1 M phosphate buffer (pH 6.8) and 1 mL of 1.5% (*w*/*v*) potassium iron tartrate. The absorbance was measured at 540 nm. The standard curve was generated with ethyl gallate.

The flavonoid content was determined by using the method described by Zhishen et al. [74]. Briefly, 1 g of fresh tissue was homogenized in methanol and ethanol (3.5:1.5, *v*/*v*). The homogenate was centrifuged at 12,000× *g* for 20 min, and the supernatant was filtered using a 0.45-μm filter membrane. Then, 1 mL of filtrate was added to a mixture containing 0.3 mL of 5% (*m*/*v*) sodium nitrite, 0.3 mL of 10% (*m*/*v*) aluminium nitrate, and 2 mL of 2 M sodium hydroxide. After 10 min of incubation, the flavonoid content was measured at 510 nm. Rutin was used to generate the standard curve.

The anthocyanin content was determined using the method of Abdel-Aal and Hucl [75]. In brief, 1 g of fresh sample was cut into 2-mm segments and transferred into a tube containing 10 mL of 0.1 M hydrochloric acid. After 4 h of incubation at 32 °C, the anthocyanin content was measured at 530 nm. Proanthocyanidin was used to make the standard curve.

### 4.14. Determination of Relative Gene Expression

Total RNA was extracted from 0.2 g of tissues using the RNAprep Pure Plant Kit (TIANGEN Biotech, Co., Ltd., Beijing, China). cDNA was synthesized with 0.1 μg of RNA using the SuperScript First-Strand Synthesis System (Thermo Fisher Scientific, Chicago, IL, USA). The gene-specific primers (Table S2) were designed according to the gene sequences, which were obtained from the National Center for Biotechnology Information (NCBI). qRT-PCR was performed using the StepOnePlus™ RealTime PCR System (Applied Biosystems, Foster City, CA, USA), and actin was used as an internal control. The relative expression of genes were calculated following the method of Shen et al. [76].

### 4.15. Statistical Analysis

Data were analyzed by using the SPSS 21.0 statistical program (IBM Inc., Chicago, IL, USA) and expressed as the mean ± standard error (*n* = 4). Analysis of variance (ANOVA) was performed, and the mean differences were evaluated by Duncan’s multiple range test at *p* < 0.05.

## 5. Conclusions

The current study demonstrated the positive role of exogenous application of melatonin in enhancing salt stress tolerance in sugar beets. Under salt stress, all methods of melatonin application enhanced growth parameters, promoted photosynthesis, improved water status, maintained ion homeostasis, and strengthened the antioxidant defense system for the removal of ROS. These might be the potential mechanisms by which melatonin mitigated the damage to sugar beets caused by salt stress. Moreover, combined root and foliar melatonin application was more effective than root or foliar application alone in improving the salt stress tolerance of sugar beets. In future studies, the mechanism by which melatonin enhances salt stress tolerance in plants should be further explored through in-depth molecular means, and multi-year experiments could be conducted under field conditions to verify the advantageous effects of melatonin.

## Data Availability

The data presented in this study are available on request from the corresponding author.

## Supplementary Materials

The following are available online at https://www.mdpi.com/article/10.3390/plants10050886/s1, Table S1: Chemical properties of the experimental soil, Table S2: Gene-specific primers used for qRT-PCR.

## References

[1] FAO (2008). Land and Plant Nutrition Management Service. http://www.fao.org/ag/agl/agll/spush/.

[2] FAO (2019). www.fao.org/faostat/en/#data.

[3] Da Silva C.J., Fontes E.P.B., Modolo L.V. (2017). Salinity-induced accumulation of endogenous H_2_S and NO is associated with modulation of the antioxidant and redox defense systems in *Nicotiana tabacum* L. cv. Havana. Plant Sci..

[4] Bor M., Ozdemir F., Turkan I. (2003). The effect of salt stress on lipid peroxidation and antioxidants in leaves of sugar beet (*Beta vulgaris* L.) and wild beet (*Beta maritima* L.). Plant Sci..

[5] Liu H., Wang Q., Yu M., Zhang Y., Wu Y., Zhang H. (2008). Transgenic salt-tolerant sugar beet (*Beta vulgaris* L.) constitutively expressing an *Arabidopsis thaliana* vacuolar Na^+^/H^+^ antiporter gene, *AtNHX3*, accumulates more soluble sugar but less salt in storage roots. Plant Cell Environ..

[6] Hossain M.S., Persicke M., ElSayed A.I., Kalinowski J., Dietz K.J. (2017). Metabolite profiling at the cellular and subcellular level reveals metabolites associated with salinity tolerance in sugar beet. J. Exp. Bot..

[7] Zhu J.K. (2001). Plant salt tolerance. Trends Plant Sci..

[8] Munns R., Tester M. (2008). Mechanisms of salinity tolerance. Annu. Rev. Plant Biol..

[9] Hasanuzzaman M., Alam M.M., Rahman A., Hasanuzzaman M., Nahar K., Fujita M. (2014). Exogenous proline and glycine betaine mediated upregulation of antioxidant defense and glyoxalase systems provides better protection against salt-induced oxidative stress in two rice (*Oryza sativa* L.) varieties. Biomed Res. Int..

[10] Per T.S., Khan N.A., Reddy P.S., Masood A., Hasanuzzaman M., Khan M.I.R., Anjum N.A. (2017). Approaches in modulating proline metabolism in plants for salt and drought stress tolerance: Phytohormones, mineral nutrients and transgenics. Plant Physiol. Biochem..

[11] Anschutz U., Becker D., Shabala S. (2014). Going beyond nutrition: Regulation of potassium homoeostasis as a common denominator of plant adaptive responses to environment. J. Plant Physiol..

[12] Chinnusamy V., Jagendorf A., Zhu J.K. (2005). Understanding and improving salt tolerance in plants. Crop Sci..

[13] Ramon Acosta-Motos J., Fernanda Ortuno M., Bernal-Vicente A., Diaz-Vivancos P., Jesus Sanchez-Blanco M., Antonio Hernandez J. (2017). Plant responses to salt stress: Adaptive mechanisms. Agronomy.

[14] Mittler R. (2002). Oxidative stress, antioxidants and stress tolerance. Trends Plant Sci..

[15] Blokhina O., Virolainen E., Fagerstedt K.V. (2003). Antioxidants, oxidative damage and oxygen deprivation stress: A review. Ann. Bot..

[16] Sharma A., Kumar V., Shahzad B., Ramakrishnan M., Sidhu G.P.S., Bali A.S., Handa N., Kapoor D., Yadav P., Khanna K. (2020). Photosynthetic response of plants under different abiotic stresses: A review. J. Plant Growth Regul..

[17] Khanna-Chopra R. (2012). Leaf senescence and abiotic stresses share reactive oxygen species-mediated chloroplast degradation. Protoplasma.

[18] Negrao S., Schmockel S.M., Tester M. (2017). Evaluating physiological responses of plants to salinity stress. Ann. Bot..

[19] Farhangi-Abriz S., Torabian S. (2017). Antioxidant enzyme and osmotic adjustment changes in bean seedlings as affected by biochar under salt stress. Ecotoxicol. Environ. Saf..

[20] Iqbal N., Umar S., Khan N.A., Khan M.I.R. (2014). A new perspective of phytohormones in salinity tolerance: Regulation of proline metabolism. Environ. Exp. Bot..

[21] Mansour M.M.F., Ali E.F. (2017). Evaluation of proline functions in saline conditions. Phytochemistry.

[22] Tester M., Davenport R. (2003). Na^+^ tolerance and Na^+^ transport in higher plants. Ann. Bot..

[23] Apse M.P., Aharon G.S., Snedden W.A., Blumwald E. (1999). Salt tolerance conferred by overexpression of a vacuolar Na^+^/H^+^ antiport in *Arabidopsis*. Science.

[24] Navrot N., Rouhier N., Gelhaye E., Jacquot J.P. (2007). Reactive oxygen species generation and antioxidant systems in plant mitochondria. Physiol. Plant..

[25] Karuppanapandian T., Moon J.-C., Kim C., Manoharan K., Kim W. (2011). Reactive oxygen species in plants: Their generation, signal transduction, and scavenging mechanisms. Aust. J. Crop Sci..

[26] Das S.K., Patra J.K., Thatoi H. (2016). Antioxidative response to abiotic and biotic stresses in mangrove plants: A review. Int. Rev. Hydrobiol..

[27] Agati G., Azzarello E., Pollastri S., Tattini M. (2012). Flavonoids as antioxidants in plants: Location and functional significance. Plant Sci..

[28] Gill S.S., Anjum N.A., Hasanuzzaman M., Gill R., Trivedi D.K., Ahmad I., Pereira E., Tuteja N. (2013). Glutathione and glutathione reductase: A boon in disguise for plant abiotic stress defense operations. Plant Physiol. Biochem..

[29] Desoky E.S.M., Merwad A.R.M., Rady M.M. (2018). Natural biostimulants improve saline soil characteristics and salt stressed-sorghum performance. Commun. Soil Sci. Plant Anal..

[30] Qin H., Li Y., Huang R. (2020). Advances and challenges in the breeding of salt-tolerant rice. Int. J. Mol. Sci..

[31] Bahcesular B., Yildirim E.D., Karacocuk M., Kulak M., Karaman S. (2020). Seed priming with melatonin effects on growth, essential oil compounds and antioxidant activity of basil (*Ocimum basilicum* L.) under salinity stress. Ind. Crop. Prod..

[32] Liu L., Liu D., Wang Z., Zou C., Wang B., Zhang H., Gai Z., Zhang P., Wang Y., Li C. (2020). Exogenous allantoin improves the salt tolerance of sugar beet by increasing putrescine metabolism and antioxidant activities. Plant Physiol. Biochem..

[33] Kubatka P., Zubor P., Busselberg D., Kwon T.K., Adamek M., Petrovic D., Opatrilova R., Gazdikova K., Caprnda M., Rodrigo L. (2018). Melatonin and breast cancer: Evidences from preclinical and human studies. Crit. Rev. Oncol. Hematol..

[34] Arnao M.B., Hernandez-Ruiz J. (2019). Melatonin: A New Plant Hormone and/or a Plant Master Regulator?. Trends Plant Sci..

[35] Tan D.X., Manchester L.C., Reiter R.J., Qi W.B., Karbownik M., Calvo J.R. (2000). Significance of melatonin in antioxidative defense system: Reactions and products. Biol. Signals Recept..

[36] Wang P., Sun X., Li C., Wei Z., Liang D., Ma F. (2013). Long-term exogenous application of melatonin delays drought-induced leaf senescence in apple. J. Pineal Res..

[37] Caputo G.A., Wadl P.A., McCarty L., Adelberg J., Jennings K.M., Cutulle M. (2020). In vitro safening of bentazon by melatonin in sweetpotato (*Ipomoea batatas*). Hortscience.

[38] Jahan M.S., Guo S., Baloch A.R., Sun J., Shu S., Wang Y., Ahammed G.J., Kabir K., Roy R. (2020). Melatonin alleviates nickel phytotoxicity by improving photosynthesis, secondary metabolism and oxidative stress tolerance in tomato seedlings. Ecotoxicol. Environ. Saf..

[39] Yan F., Wei H., Li W., Liu Z., Tang S., Chen L., Ding C., Jiang Y., Ding Y., Li G. (2020). Melatonin improves K^+^ and Na^+^ homeostasis in rice under salt stress by mediated nitric oxide. Ecotoxicol. Environ. Saf..

[40] Tousi S., Zoufan P., Ghahfarrokhie A.R. (2020). Alleviation of cadmium-induced phytotoxicity and growth improvement by exogenous melatonin pretreatment in mallow (*Malva parviflora*) plants. Ecotoxicol. Environ. Saf..

[41] Zhang T.G., Shi Z.F., Zhang X.H., Zheng S., Wang J., Mo J.N. (2020). Alleviating effects of exogenous melatonin on salt stress in cucumber. Sci. Hortic..

[42] Li J., Yuan F., Liu Y., Zhang M., Liu Y., Zhao Y., Wang B., Chen M. (2020). Exogenous melatonin enhances salt secretion from salt glands by upregulating the expression of ion transporter and vesicle transport genes in *Limonium bicolor*. Bmc Plant Biol..

[43] Ali M., Kamran M., Abbasi G.H., Saleem M.H., Ahmad S., Parveen A., Malik Z., Afzal S., Ahmar S., Dawar K.M. (2020). Melatonin-induced salinity tolerance by ameliorating osmotic and oxidative stress in the seedlings of two tomato (*Solanum lycopersicum* L.) cultivars. J. Plant Growth Regul..

[44] Ren J., Ye J., Yin L., Li G., Deng X., Wang S. (2020). Exogenous melatonin improves salt tolerance by mitigating osmotic, ion, and oxidative stresses in maize seedlings. Agronomy.

[45] Kaur H., Bhatla S.C. (2016). Melatonin and nitric oxide modulate glutathione content and glutathione reductase activity in sunflower seedling cotyledons accompanying salt stress. Nitric Oxide.

[46] Kaya C., Higgs D., Ashraf M., Alyemeni M.N., Ahmad P. (2020). Integrative roles of nitric oxide and hydrogen sulfide in melatonin-induced tolerance of pepper (*Capsicum annuum* L.) plants to iron deficiency and salt stress alone or in combination. Physiol. Plant..

[47] Wang D., Chen Q., Chen W., Guo Q., Xia Y., Wang S., Jing D., Liang G. (2021). Physiological and transcription analyses reveal the regulatory mechanism of melatonin in inducing drought resistance in loquat (*Eriobotrya japonica* Lindl.) seedlings. Environ. Exp. Bot..

[48] Kamiab F. (2020). Exogenous melatonin mitigates the salinity damages and improves the growth of pistachio under salinity stress. J. Plant Nutr..

[49] Borisova-Mubarakshina M.M., Ivanov B.N., Vetoshkina D.V., Lubimov V.Y., Fedorchuk T.P., Naydov I.A., Kozuleva M.A., Rudenko N.N., Dall’Osto L., Cazzaniga S. (2015). Long-term acclimatory response to excess excitation energy: Evidence for a role of hydrogen peroxide in the regulation of photosystem II antenna size. J. Exp. Bot..

[50] Yin Z.P., Lu J.Z., Meng S.D., Liu Y.L., Mostafa I., Qi M.F., Li T.L. (2019). Exogenous melatonin improves salt tolerance in tomato by regulating photosynthetic electron flux and the ascorbate-glutathione cycle. J. Plant Interact..

[51] Ding F., Liu B., Zhang S. (2017). Exogenous melatonin ameliorates cold-induced damage in tomato plants. Sci. Hortic..

[52] Qiao Y., Ren J., Yin L., Liu Y., Deng X., Liu P., Wang S. (2020). Exogenous melatonin alleviates PEG-induced short-term water deficiency in maize by increasing hydraulic conductance. BMC Plant Biol..

[53] Farouk S., Elhindi K.M., Alotaibi M.A. (2020). Silicon supplementation mitigates salinity stress on *Ocimum basilicum* L. via improving water balance, ion homeostasis, and antioxidant defense system. Ecotoxicol. Environ. Saf..

[54] Leopoldini M., Russo N., Chiodo S., Toscano M. (2006). Iron chelation by the powerful antioxidant flavonoid quercetin. J. Agric. Food. Chem..

[55] Mukherjee S. (2018). Novel perspectives on the molecular crosstalk mechanisms of serotonin and melatonin in plants. Plant Physiol. Biochem..

[56] Chung R.S., Chen C.C., Ng L.T. (2010). Nitrogen fertilization affects the growth performance, betaine and polysaccharide concentrations of *Lycium barbarum*. Ind. Crop. Prod..

[57] Bates L.S., Waldren R.P., Teare I.D. (1973). Rapid determination of free proline for water-stress studies. Plant Soil..

[58] Spiro R.G. (1966). Analysis of sugars found in glycoproteins. Methods Enzymol..

[59] Chen Y.E., Mao J.J., Sun L.Q., Huang B., Ding C.B., Gu Y., Liao J.Q., Hu C., Zhang Z.W., Yuan S. (2018). Exogenous melatonin enhances salt stress tolerance in maize seedlings by improving antioxidant and photosynthetic capacity. Physiol. Plant..

[60] Yuan F., Yang H., Xue Y., Kong D., Ye R., Li C., Zhang J., Theprungsirikul L., Shrift T., Krichilsky B. (2014). *OSCA1* mediates osmotic-stress-evoked Ca^2+^ increases vital for osmosensing in *Arabidopsis*. Nature.

[61] Storey R. (1995). Salt Tolerance, ion relations and the effect of root medium on the response of citrus to salinity. Funct. Plant Biol..

[62] Kong X., Luo Z., Dong H., Eneji A.E., Li W. (2012). Effects of non-uniform root zone salinity on water use, Na^+^ recirculation, and Na^+^ and H^+^ flux in cotton. J. Exp. Bot..

[63] Zhang W., Yu X., Li M., Lang D., Zhang X., Xie Z. (2018). Silicon promotes growth and root yield of glycyrrhiza uralensis under salt and drought stresses through enhancing osmotic adjustment and regulating antioxidant metabolism. Crop Prot..

[64] Velikova V., Yordanov I., Edreva A. (2000). Oxidative stress and some antioxidant systems in acid rain-treated bean plants. Plant Sci..

[65] Rajinder S.D., Pamela P.D., Trevor A.T. (1981). Leaf senescense: Correlated with increased levels of membrane permeability and lipid peroxidation, and decreased levels of superoxide dismutase and catalase. J. Exp. Bot..

[66] Dionisio-Sese M.L., Tobita S. (1998). Antioxidant responses of rice seedlings to salinity stress. Plant Sci..

[67] Giannopolitis C.N., Ries S.K. (1977). Superoxide dismutases: I. Occurrence in higher plants. Plant Physiol..

[68] He J., Ren Y., Chen X., Chen H. (2014). Protective roles of nitric oxide on seed germination and seedling growth of rice (*Oryza sativa* L.) under cadmium stress. Ecotoxicol. Environ. Saf..

[69] Nabih I., el-Wassimy M.T., Metri J. (1968). Structure and activity in molluscicides. II. Activity assary of catalase and peroxidase in snails. J. Pharm Sci..

[70] Fielding J.L., Hall J.L. (1978). A biochemical and cytochemical study of peroxidase activity in poots of Pisum sativum. J. Exp. Bot..

[71] Logan B.A., Grace S.C., Adams Iii W.W., Demmig-Adams B. (1998). Seasonal differences in xanthophyll cycle characteristics and antioxidants in Mahonia repens growing in different light environments. Oecologia.

[72] Griffith O.W. (1980). Determination of glutathione and glutathione disulfide using glutathione reductase and 2-vinylpyridine. Anal. Biochem..

[73] Singleton V., Rossi J.A. (1964). Colorimetry of total phenolics with phosphomolybdic-phosphotungstic acid reagents. Am. J. Enol. Vitic..

[74] Zhishen J., Mengcheng T., Jianming W. (1999). The determination of flavonoid contents in mulberry and their scavenging effects on superoxide radicals. Food Chem..

[75] Abdel-Aal E.S.M., Hucl P.A. (1999). A Rapid Method for quantifying total anthocyanins in blue aleurone and purple pericarp wheats. Cereal Chem..

[76] Shen J.L., Wang Y., Shu S., Jahan M.S., Zhong M., Wu J.Q., Sun J., Guo S.R. (2019). Exogenous putrescine regulates leaf starch overaccumulation in cucumber under salt stress. Sci. Hortic..

