# Supplementary material for: Beneficial Effects of Exogenous Melatonin on Overcoming Salt Stress in Sugar Beets (Beta vulgaris L.)
Source: Plants (Basel). 2021 Apr 28;10(5):886. doi: 10.3390/plants10050886 (PMC8146524; doi:10.3390/plants10050886)
Supplement: Supplementary file 1 [file plants-10-00886-s001.zip › plants-1191715-supplementary.pdf]

Table S1

Chemical properties of the experimental soil.

pH (1:2.5 water extract)	Electrical conductivity ($dS\ m^{-1}$)	Available nitrogen ($mg\ kg^{-1}$)	Available phosphorus ($mg\ kg^{-1}$)	Available potassium ($mg\ kg^{-1}$)	Organic matter ($g\ kg^{-1}$)
7.35	0.28	133.12	24.11	173.58	32.36

Table S2. Gene-specific primers used for qRT-PCR.

Gene full name	Gene abbreviation	Forward primer	Reverse primer
Chlorophyll synthase	*CHLG*	GATTGGTACGACCGAGAA	ACAACCCGCTACAGATAA
Protochlorophyllide oxidoreductase	*POR*	CTGCGAAAGGGAAATGTCGTG	TCGAGGTGCATGATGCTGTAA
Chlorophyllide a oxygenase	*CAO*	GGAACTGGTATGGTCAAGGC	AGTCTCATGGATAACCGCAG
Bataine aldehyde dehydrogenase	*BADH*	CTCTAGACACCCAAAGCCCAAGCTC	GGTACCCACACGACTTCAGCACATG
Delta'-pyrroline-5-carboxylate synthase	*P5CS*	ATGGACGAAGTGGATCGTTCTCG	TTAGGGCTCAATTGGGAGGTCC
High-affinity potassium transporter	*HKT1*	CTCACTGCGACTGATGTTCCTGGGC	GCAAGTTGTCATTTGGTGGGCTGGTC
Na^+/H^+ antiporter	*NHX1*	GCAATCCAGAGCTTCGACCTTACGC	AGCACCTTGCTTGGAGCGATGACAG
H^+-ATPase	*HA*	AGCCTTTGTCATTGCCCAGCTG	ACCAGCCCAACCCCATCCAATC
H^+-PPase	*HP*	TCACTAGTTTCCGACGGTTG	CTTCCGGAATAACACAAGCA
Superoxide dismutase	*SOD*	TGGAATGTCACTGTTGGGGA	ACAACTGCCCTACCAATGACA
Peroxidase	*POD*	GTCAGGAACCACCTCAAG	CACCAGACAGATAAACGGA
Catalase	*CAT*	CAGATCTATGGATCCTTACAAGTA	CAAGCTTCATGGTTGGTCTTATGTTAA
Ascorbate peroxidase	*APX*	GATTTCCATCAGCTTGCTGG	GGTCAGTTAAACCCATCTGC
Monodehydroascorbate reductase	*MDHAR*	CCAGCGCCAACACAAGTATG	GCTGGATATGCTGCTAGAACT
Dehydroascorbate reductase	*DHAR*	AAGAAGGTGCCCTACCAGATG	TAGATGCAGTAAAGAACCTGAC
Glutathione reductase	*GR*	ATGTCGAGGAAGATGCTAATTG	CTACAGATTTGTCTTTGGTTTGGTA
Glutathione S-transferase	*GST*	ATGGCGAATGAGGTGAAATTGC	TTACGCCTTACCAAACCTTGAC
Glutathione peroxidase	*GPX*	TCAAACTCCCCGTCTTATGG	GCTCCAACTGAATCCTTGC
Phenylalanine ammonia-lyase	*PAL*	CCTCCTCCTCCTTCGGTTTG	GCCATGAATGTCACCGCAGA
Chalcone synthase	*CHS*	GAATCCTGAAGCCGCCACGAAAG	CCTCCAGTGCTCACCGTAGACAG
Flavonol synthase	*FLS*	ACCATAGCCAAGCCTGCAAA	TGAAACAAGTGGTCCACCCAC
Anthocyanidin synthase	*ANS*	AATGCTAGTGGACAGCTTGAG	TTGACCAGTCCCATGTGAAG
Actin		CACACCAGATGAAGGCCGT	CCCTGAAGACCGTGCCAT
